# Supplementary material for: Brain Proteome and Behavioural Analysis in Wild Type, BDNF+/− and BDNF−/− Adult Zebrafish (Danio rerio) Exposed to Two Different Temperatures
Source: Int J Mol Sci. 2022 May 17;23(10):5606. doi: 10.3390/ijms23105606 (PMC9146406; doi:10.3390/ijms23105606)
Supplement: Supplementary file 1 [file ijms-23-05606-s001.zip › ijms-1658523-supplementary/Figure S2.pdf]

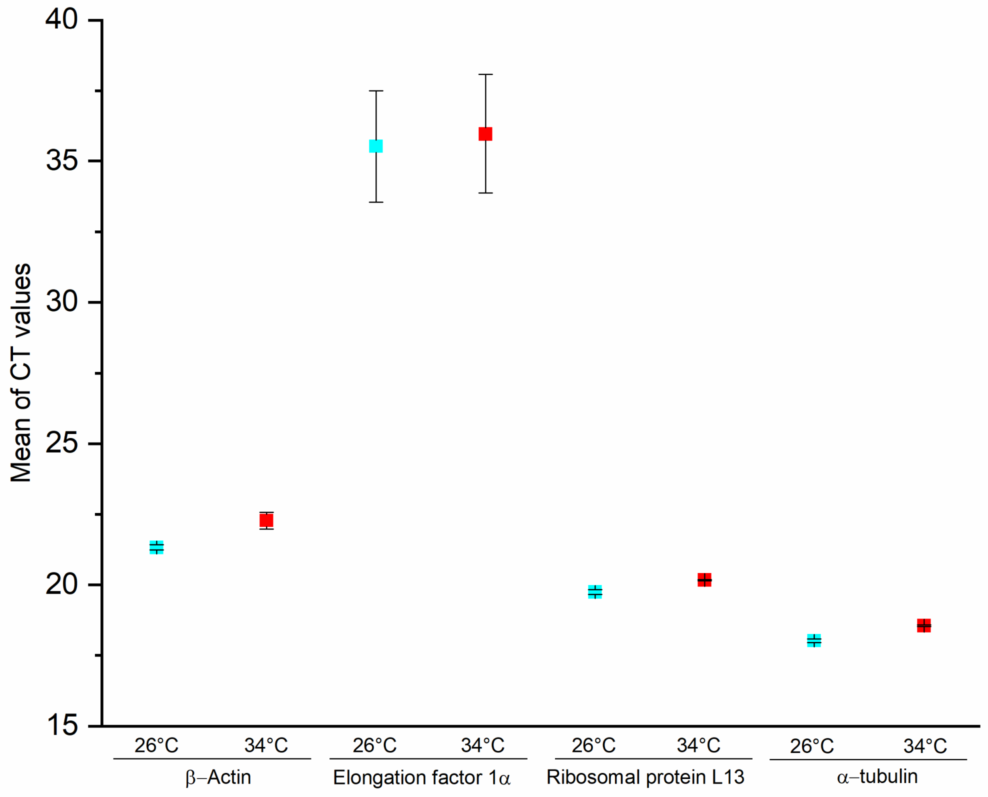

Figure S2. Real-time PCR results and Ct values for evaluating the stability of housekeeping genes (actin, elongation factor 1 a, ribosomal protein L13a and tubulin) in the brain of adult WT zebrafish kept at 26° C or 34° C for 21 days. . Experiments were performed in triplicate and results are expressed as mean ± SD. N=12. Blue and red colours refer to 26° C and 34° C, respectively.
